# Behavioral and neuronal study of inhibition of return in barn owls

**DOI:** 10.1038/s41598-020-64197-9

**Published:** 2020-04-29

**Authors:** Tidhar Lev-Ari, Yael Zahar, Arpit Agarwal, Yoram Gutfreund

**Affiliations:** Department of Neuroscience, Ruth and Bruce Rappaport Faculty of Medicine and Research Institute, Technion – Israel Institute of Technology, Haifa, 31096 Israel

**Keywords:** Attention, Sensory processing

## Abstract

Inhibition of return (IOR) is the reduction of detection speed and/or detection accuracy of a target in a recently attended location. This phenomenon, which has been discovered and studied thoroughly in humans, is believed to reflect a brain mechanism for controlling the allocation of spatial attention in a manner that enhances efficient search. Findings showing that IOR is robust, apparent at a very early age and seemingly dependent on midbrain activity suggest that IOR is a universal attentional mechanism in vertebrates. However, studies in non-mammalian species are scarce. To explore this hypothesis comparatively, we tested for IOR in barn owls (*Tyto alba*) using the classical Posner cueing paradigm. Two barn owls were trained to initiate a trial by fixating on the center of a computer screen and then turning their gaze to the location of a target. A short, non-informative cue appeared before the target, either at a location predicting the target (valid) or a location not predicting the target (invalid). In one barn owl, the response times (RT) to the valid targets compared to the invalid targets shifted from facilitation (lower RTs) to inhibition (higher RTs) when increasing the time lag between the cue and the target. The second owl mostly failed to maintain fixation and responded to the cue before the target onset. However, when including in the analysis only the trials in which the owl maintained fixation, an inhibition in the valid trials could be detected. To search for the neural correlates of IOR, we recorded multiunit responses in the optic tectum (OT) of four head-fixed owls passively viewing a cueing paradigm as in the behavioral experiments. At short cue to target lags (<100 ms), neural responses to the target in the receptive field (RF) were usually enhanced if the cue appeared earlier inside the RF (valid) and were suppressed if the cue appeared earlier outside the RF (invalid). This was reversed at longer lags: neural responses were suppressed in the valid conditions and were unaffected in the invalid conditions. The findings support the notion that IOR is a basic mechanism in the evolution of vertebrate behavior and suggest that the effect appears as a result of the interaction between lateral and forward inhibition in the tectal circuitry.

## Introduction

Efficient ecological search is a major evolutionary drive leading to sophisticated mechanisms for searching the environment for behaviorally relevant elements such as food, threats or mates^1^. Psychophysical experiments in humans have revealed the phenomenon of inhibition of return (IOR). In classical IOR experiments (known as the Posner cueing paradigm^2,3^), subjects are required to find a target that can either be in a previously cued location (valid trial) or in a location different from the cued location (invalid trial). When the cue to target onset asynchrony (CTOA) is short (usually <100 ms), response times (RT) to detect targets in valid trials tend to be faster compared to RTs in invalid trials. However, when the CTOA is longer (usually >300 ms), the order changes: RTs in valid trials tend to be slower compared to invalid trials^4^.

IOR is generally thought to be a guiding mechanism for orienting to novel locations by reducing the chances of re-inspecting previously attended locations^5,6^. A salient cue reflexibly attracts attention to the cued location, but if nothing important appears there, then filing that location as one to avoid will make further search processes more efficient. The inhibitory after-effect of IOR may be the mechanism for this filing^7^. Thus, our visual search is governed by two processes: one is attentional capture that explains the facilitation of responses to the valid targets at short CTOAs^8,9^; the second is IOR that explains the inhibition of responses to valid targets at longer CTOAs^3,10^. In humans, IOR based on spatial cueing was shown to be a robust phenomenon^11,12^ detected even in newborns^13,14^.

Several studies in brain-damaged individuals point to the superior colliculus (SC) as being a source of inhibition of return^15,16^. The SC is an anatomically conserved structure in vertebrate evolution known as the optic tectum (OT) in non-mammalian vertebrates^17^. Recent findings suggest that the function of the OT in orientation and attention is conserved across vertebrates all the way to primates^18,19^. The functional localization of IOR in humans to an archaic brain structure suggests that the phenomenon may be a universal mechanism that evolved early in evolution to support an efficient search. Support for this notion includes recent studies demonstrating that the basic features of IOR are found in the archer fish^20,21^. However, studies in other non-mammalian vertebrates are scarce.

In this study, we addressed two questions: 1) Do barn owls possess behavioral responses akin to IOR? 2) Can we find neural correlates of IOR in the responses of the barn owl’s OT? Owls rely both on visual and auditory inputs for rapid detection of small prey items in highly cluttered, dimmed and noisy environments, conditions that are challenging to any attentional system. Barn owls (*Tyto alba*) have been shown to possess well-developed bottom-up attentional mechanisms, including cueing effects, attentional capture and pop-out perception^22–24^. Moreover, the OT of barn owls has been studied thoroughly, providing a system that is well characterized and accessible for electrophysiological analysis^25,26^.

To facilitate comparison, we tested two barn owls in a Posner cueing task commonly used in humans and monkeys^27,28^. Although the two owls showed behavioral differences, the responses were comparable to the results measured from human subjects, suggesting the existence of basic IOR in barn owls. In a parallel experiment, we measured neural responses in the OT of owls, passively viewing a cueing paradigm as in the behavioral experiments. Neural responses were stronger for the validly cued targets at short time lags and stronger for the invalidly cued targets at longer time lags. These results support the notion that IOR is a basic mechanism in the evolution of vertebrate behavior and suggest that the effect appears as a result of the interaction between lateral and forward inhibition in tectal circuitry.

## Methods

### Animals

Six adult barn owls were used in this study. The owls were hatched and raised in captivity and housed in aviaries equipped with perching spots and brooding boxes. All procedures were in accordance with the guidelines and were approved by the Technion’s Institutional Animal Care and Use Committee. All surgical procedures were performed under isoflurane anesthesia, and the animals were sedated with a mixture of oxygen and nitrous oxide in all recording sessions. No painful procedures were carried out during the recording sessions.

### Behavioral experiments

Behavioral measurements were conducted from hand-raised barn owls that were pre-trained in previous projects to initiate a trial by fixating on a red dot at the center of a computer screen and turning their gaze to a circular target on the screen^22,29^. In the current task, after fixation was achieved, a cue (black rectangle 4 × 4 cm) appeared for 33 ms (two frames). Cue location was chosen randomly either 6.3 cm to the right of the fixation point or 6.3 cm to the left with equal probability. Following the cue, a target appeared (black circle of 3 cm diameter) at one of three possible CTOAs (66, 433, 633 ms). The target position was chosen randomly either to the left or right of the center (±6.3 cm). Thus, the cue was uninformative, i.e., in about 50% of the trials, the target appeared at the location of the cue (valid trials) and 50% at the opposite location (invalid trials). The screen background was maintained at a mid-gray level.

**Figure 1:**
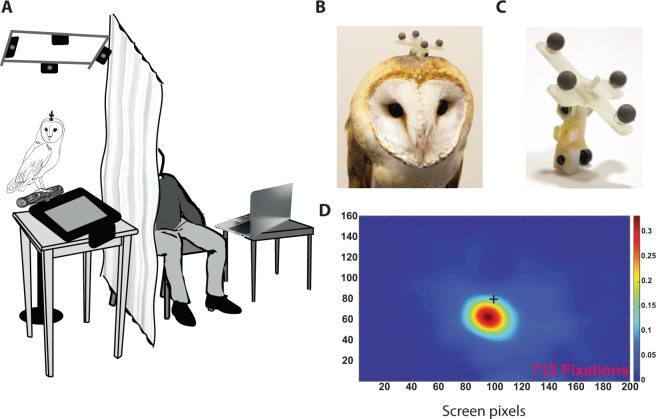
The experimental set-up. (**A)** An illustration of the behavioral setup. (**B**) An owl with the IR reflectors device mounted in-place. (**C**) A close view of the IR reflectors device with the attachment unit. (**D**) A heat map showing the rate of fixation points on the screen relative to the position of a circular black target on the screen (black plus). Heat map was constructed from 712 fixation points.

For the experiment, the owl was placed on a perch in a dimly lit room with the computer screen (17” AccuSync, NCE, Illinois, USA, 1280/1024 pixels at a refresh rate of 60 Hz) that was facing upwards in a pecking range below the owl (Fig. 1A and Supplementary video). Owls typically searched the screen from a distance of about 25 cm. Four high-speed infrared cameras were used to track the 3D positions of four IR reflectors rigidly attached to the owl’s head (OptiTrack system, Oregon, USA). The markers were attached to the head with a 3D printed attachment unit designed to maintain a fixed and reproducible relationship between the markers and the head (Fig. 1B).

Recordings of the markers’ positions were made using Matlab software (Mathworks) with the real-time markers’ positions streamed from the Motive Software (NaturalPoint, Inc. Optitrack) using the NatNet Matlab Client (Optitrack) at 120 frame/s. The four IR markers were arranged so that the back and front markers created a line that was close to parallel to the owl’s line of sight. The left and right markers were symmetrically distant from both sides of the center line and were positioned closer to the front marker to allow differentiating between front and back (Fig. 1C). We used 3D coordinate geometry to calculate the equation of the line passing through the front and back points by calculating directions vectors d = (l, m, n):

$$\frac{x-{x}_{1}}{l}=\frac{y-{y}_{1}}{m}=\frac{z-{z}_{1}}{n}$$

where (x, y, z) are the XYZ coordinates of one of the reflectors, and the direction vector is calculated as the difference between two points on the line.

Additional markers were positioned at three corners of the screen (points A, B and C) to calculate the screen plane. We calculated the normal vector,

$\overrightarrow{n}=\overrightarrow{AB\,}\,$ × $\overrightarrow{AC}$ and from this, the equation of the plane:

$${n}_{x}x+{n}_{y}y+{n}_{z}z-(\overrightarrow{n}\cdot \overrightarrow{A})=0$$

Next, using the parametric form of the line of sight, we calculated the point of intersection (P) of the line of sight with the screen plane. Using the dimensions of the screen, we calculated if the gaze lays on the screen and its distance from the top left corner using the formula:

$\frac{\overrightarrow{|BP|}\times \overrightarrow{|BA|}\,}{\overrightarrow{|BA|}\,}$ for horizontal direction and $\frac{\overrightarrow{|BP|}\times \overrightarrow{|BC|}\,}{\overrightarrow{|BC|}\,}$ for vertical direction. This distance was then converted to screen pixels.

Since barn owls do not move their eyes in their orbits, a trajectory on the screen of a direct line emerging from the rigid body defined by the markers on the owl’s head can provide a reliable estimation of the owl’s gaze point on the screen^30^. Initially, the position of the gaze center (functional fovea) relative to the line projected by the head markers was calibrated for each owl by allowing the owl to fixate on a black dot appearing at random locations on the screen. Figure 1D shows the probability of the intersection point relative to the position of the target (calculated from 712 fixation points on the screen). The result shows a single high probability spot, reflecting the owl’s spontaneous tendency to gaze at the target and the existence of a single functional fovea in owls^31^. The distance in screen pixels between the intersection point and the target was used to correct the intersection point online to the true gaze position on the screen. A GUI was designed so that the experimenter could view on a laptop the owl’s gaze relative to targets on the screen in real time. The program also reported in real time a successful gaze on the target for reward purposes.

**Figure 2:**
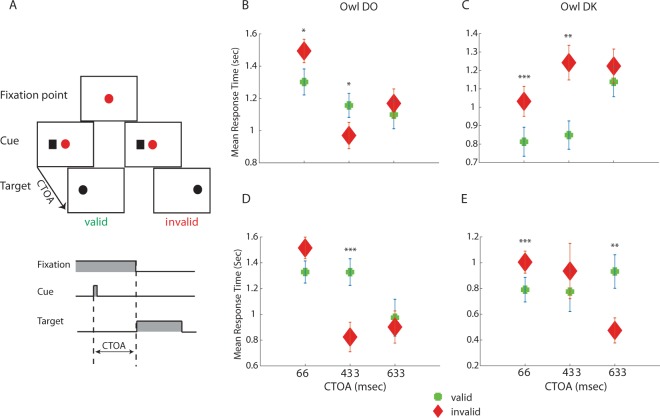
Average response times to the target as a function of the CTOA. (**A)** An illustration of the cueing paradigm. Fixation point - red circle; non-informative cue - black rectangle; target - black circle. Time axis of a trial is shown in the lower inset. gray area designate the on period. (**B)** The average response times of all successful trials as a function of the CTOA, shown seperately for valid trials (circles) and for invalid trials (diamonds). Error bars designate SEMs. Behavioral data from owl DO. (**C)** same as in B but showing behavioral data of owl DK. (**D,E**) Same as(**B,C**) but showing data from trials in-which the gaze point was maintained on red circle (central fixation point) until the appearance of the target.

A fixation was considered to be on the target if any area of the target appeared inside a circular area of 2 cm diameter surrounding the gaze point on the screen and was maintained there for at least 150 ms. In each trial, the time from stimulus onset to the first fixation on the target (response time) was registered. The owls were rewarded for gazing at the target within a time window of 3 s after the appearance of the target. Rewards were small chunks of chicken meat given with forceps held just above the target position (see Supplementary video). A trial was considered a failure if the owl did not fixate on the target during the 3 s of target display and was not included in the analysis. Trials in which the gaze position stayed inside a 3 × 3 cm zone around the central fixation point during the CTOA (maintained fixation) were analyzed separately (Fig. 2D,E).

The owls performed about 50 trials a day. We tested the owls on consecutive days until they reached more than 85 repetitions of each condition (a total of 592 and 539 repetitions for owls DK and DO, respectively). Statistical differences between responses to different stimuli were assessed using the Mann-Whitney U test.

### Human experiments

Fifteen naïve participants (average age 34 years, 10 females and 5 males) were seated in front of a computer screen (22″ S22E450, Samsung, 1680/1050 pixels at a refresh rate of 60 Hz). All of the participants had normal or corrected to normal vision. All methods were in accordance with the relevant guidelines and regulations. All experimental protocols were approved by the Technion’s Institutional Review Board and Human Subject Protection. At the beginning of the experiment the participants received instructions about the task and the purpose of the study and provided an informed consent to participate in the study. All subjects were above 18 years old. First, participants performed a 10-trial practice block and then the experimenter left the room and the participant was left to control the trials by himself. For the experiment the participant viewed the computer screen at a distance of ~50 cm in a quiet office. All of the visual displays were computed using the Matlab psych toolbox. The stimuli were similar to those presented to the owls in the behavioral experiment (target and cue sizes, durations, CTOAs and locations on screen). The participants performed 480 experimental trials in one session, 80 for each of the six different conditions (three CTOAs x valid and invalid). The subjects reported target detection by pressing ‘m’ on the keyboard for a target on the right or ‘z’ for a target on the left. Trials in which participants responded incorrectly were excluded from the analyses (<1%).

### Electrophysiological procedures

Electrophysiological procedures in this paper were done as described in Dutta *et al.*^32^ and Zahar *et al.*^29^. The four owls were prepared for repeated electrophysiological recording sessions in a single surgical procedure: the birds were anesthetized with 2% isoflurane in a 4:5 mixture of nitrous oxide and oxygen. Lidocaine (Lidocaine HCl 2% and Epinephrine) was injected locally at the incision site. A craniotomy of 1 cm diameter was performed 0.6 cm lateral to the midline and 1.7 cm anterior from the anterior tip of attachment of dorsal neck muscles to the skull. A recording chamber was then cemented to the skull (C&B Metabond adhesive) over the craniotomy. The chamber was filled with chloramphenicol ointment (5%) and sealed with a cap. The bird was allowed to recover overnight and was returned to its aviary.

At the beginning of each electrophysiological session, the owl was anesthetized briefly with isoflurane (2%) and nitrous oxide in oxygen (4:5). Once anesthetized, the owl was wrapped in a holding jacket and positioned in a stereotaxic apparatus inside a double-walled, sound-attenuating chamber (internal size – 2.05 × 1.7 × 1.95 m). The head was fixed to the apparatus after aligning the visual axis using retinal landmarks (as described in^33^). Once the bird was fixed, isoflurane was removed and the bird was maintained on a steady mixture of nitrous oxide and oxygen (4:5). The cover of the recording chamber was removed and a tungsten, epoxy-coated electrode (0.5–1.5 MΩ; Alpha-Omega, Nazareth, Israel) was driven using a motorized manipulator. Since eye movements in barn owls are limited to a range that is smaller than ±2°^34^, we did not immobilize or control eye movements. The recorded electrical signal was amplified, digitized, filtered (313–5,000 Hz) and stored using the AlphaLab SnR system (Alpha Omega, Nazareth, Israel). A threshold was set online in each experiment to select the larger units in the recording sites, isolating action potentials from a small cluster of neurons (multi-unit recording). At the end of each recording session, the recording chamber was treated with chloramphenicol ointment (5%) and closed. The owl was then returned to its home flying cage for at least one week before the next recording session.

Identifying the location of the recording site was based on stereotaxic coordinates and expected physiological properties: the OT was recognized by characteristic bursting activity and spatially restricted visual and auditory receptive fields. Position within the OT was determined based on the location of the visual receptive field (RF). The intermediate layers of the OT were located beneath the bursty layers and identified based on a transition from bursty activity to regular firing^35^. Once reaching the intermediate layers, the electrode was advanced in small steps to search for sites with clear units and visual responses. Several recording sites (10–20) were collected on each experimental day along multiple electrode penetrations. Recording sites were separated by at least 300 µm. All recording sites were from the anterior part of the left OT having visual receptive fields ranging between −20° to +10° in azimuth and −20° to +20° in elevation (minus sign designates left in azimuth and down in elevation).

### Visual stimulation

Computer-generated visual displays were projected (EB-X31 projector, Epson, Japan; 60 Hz refresh rate) on a large calibrated white screen positioned 1.5 meters in front of the owl. The size of the projected area was 150 ×115 cm corresponding to about 53° × 42°. Stimuli were generated and presented by custom-written codes with Psychtoolbox running in Matlab^36^. The background screen was gray with a luminance of about 17 cd/m^2^. During the experiment, the location of the RF was first identified by manually moving a black dot on the screen. Then a detailed span map was constructed to map the RF’s area by presenting a black dot for 200 ms at random locations and in 3° jumps across ±10° in elevation and in an azimuth from the manually determined RF location. The cue and target were black circles (corresponding to 3° in diameter). The duration of the target dot was 100 ms. The duration of the cue was 33 ms (Fig. 4) or 100 ms (Figs. 5 and 6). In each trial, the cue first appeared randomly in one of two positions: inside the RF (valid trial); or outside the RF (invalid trials). The target appeared later, inside the RF in all trials. The position of the cue outside the RF was generally 20° to the left of the center of the RF (see inset in Fig. 4A), however, in some recording sites the RF was close to the left edge of the screen, in which case, the cue was positioned 20° to the right of the RF. To reduce adaptation, stimulus positions were jittered across trials ±0.75° in elevation and ±0.75° in azimuth. Different CTOAs, valid and invalid, trials were interleaved randomly between trials. The inter-trial interval was 2 s. Each stimulus condition was repeated 10 times.

Responses to a visual stimulus were calculated as the number of spikes in a given time window after the stimulus onset minus the number of spikes during the same period of time immediately before the cue onset (baseline activity). The duration of the time window for spike count was 400 ms, starting from the onset of the target. To observe the time course of the response, we generated post-stimulus time histograms (PSTHs) with 15 ms time bins. PSTHs were normalized to the maximum value achieved in each experiment and averaged across the population.

## Results

### Behavioral experiments

Two hand-raised female barn owls (DO and DK) of about three years of age were used to study the behavioral responses. Overall success rates for fixating the target within the 3-second period exceeded 80% (82%, 486/592 and 85%, 430/539 in owls DK and DO, respectively). In both owls, when the CTOA was short (66 ms), the average RT in the valid trials was faster (Fig. 2B,C, Wilcoxon sign rank test; z = −3.991, n = 91 valid trials and 84 invalid trials, p < 0.001 and z = −1.997, n = 82 valid trials and 77 invalid trials, p = 0.04 in owls DK and DO, respectively). When the CTOA was longer (433 ms), the average RT in owl DO in the valid trials was slower compared to the invalid trials, consistent with an inhibition of return (Fig. 2B, Wilcoxon sign rank test, z = 2.355, n = 89 valid trials and 91 invalid trials, p = 0.01), whereas, the average RT in owl DK in the valid trials was faster compared to the invalid trials in the 433 ms CTOA (Fig. 2C, Wilcoxon sign rank test, z = −3.16, p = 0.0015). The RTs of both owls did not differ significantly between valid and invalid trials in the 633 ms CTOA (Fig. 2B,C, Wilcoxon sign rank test; z = −0.647, n = 82 valid trials and 73 invalid trials, p = 0.647 and z = −0.479, n = 73 valid trials and 75 invalid trials, p = 0.631, in owls DK and DO, respectively). Thus, when taking all successful trials into account, owl DO but not owl DK showed consistent results with an IOR effect. However, the owls in this experiment were not trained to maintain fixation during the CTOA until the target appears, unlike what is commonly done in primates experiments^37,38^. Thus, in a substantial number of trials, the owls moved their gaze from the fixation point before the target appeared (in 38% and 56% of total trials for owls DO and DK, respectively), particularly in the long CTOAs the owls occasionally shifted their gaze towards the cue before the target. This behavioral glitch is expected to bias RTs to be faster in the valid trials (because in some cases the gaze is already pointing towards the target when it is initiated), particularly in the long CTOAs, and thus possibly masking IOR effects.

To control for this, we selected the trials in which the fixation was maintained on or near the center until the target appearance (62% and 44% of total trials for owls DO and DK, respectively). For owl DO, the adjusted analysis maintained the pattern of IOR in the 433 ms CTOA, i.e., slower RTs in the valid trials compared to the invalid trials (Fig. 2D, Wilcoxon sign rank test; CTOA 66 ms, z = −1.692, n = 76 valid trials and 64 invalid trials, p = 0.09; CTOA 433 ms, z = 3.733, n = 38 valid trials and 41 invalid trials, p < 0.001; CTOA 633 ms, z = 0.003, n = 27 valid trials and 31 invalid trials, p = 0.975). In owl DK, the corrected analysis showed a switch to faster RTs in the invalid trials in the 633 ms CTOA (Fig. 2E, Wilcoxon sign rank test; CTOA 66 ms, z = −3.826, n = 65 valid trials and 74 invalid trials, p < 0.001; CTOA 433 ms, z = 0.454, n = 16 valid trials and 15 invalid trials, p = 0.649; CTOA 633 ms, z = 2.546, n = 31 valid trials and 11 invalid trials, p = 0.01). Thus, although the two owls showed variable behaviors, our results suggest a cueing effect akin to IOR.

**Figure 3:**
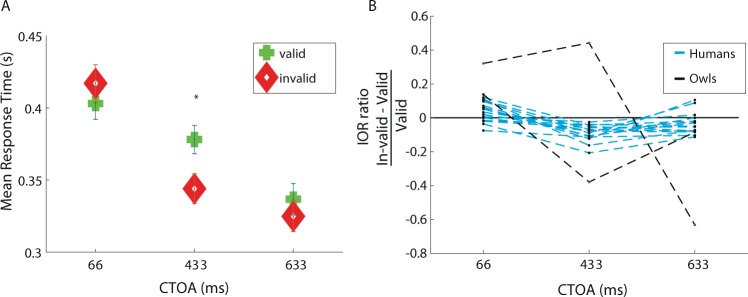
Summary of results from human participants. (**A)** The mean response time as a function of the CTOA shown seperately for valid trials (green pluss) and for invalid trials (red diamonds). Error bars designate the SEM. (**B)** The IOR ratio (invalid RT - valid RT divided by the valid RT) as a function of the CTOA, shown seperately for each of the 15 humans subjects (blue dashed lines) and for the the two owls (black dashed lines).

To facilitate comparison with humans, we tested human subjects employing the same paradigm used in owls. The difference from the experiment with owls was that the human subjects reported the location of the target by pressing left or right buttons, and that for each participant, all data were collected in a single session. The average RTs of the means of 15 subjects are shown in Fig. 3A. In the short 66 ms CTOA, responses in the valid trials were faster, but not significantly different, compared to the invalid trials (Wilcoxon sign rank test, z = −0.829, p = 0.406). Significant IOR was observed in the 433 ms CTOA (Wilcoxon sign rank test, z = 2.281, p = 0.02). In the 633 ms CTOA, RTs of invalid and valid trials were again not significantly different (Wilcoxon sign rank test, z = 1.078, p = 0.280). Thus, the IOR effect at the group level was evident, however, variability among individuals was apparent with some participants responding faster in the invalid trials, no matter what the CTOA was (Fig. 3B).

### Electrophysiological experiments

**Figure 4:**
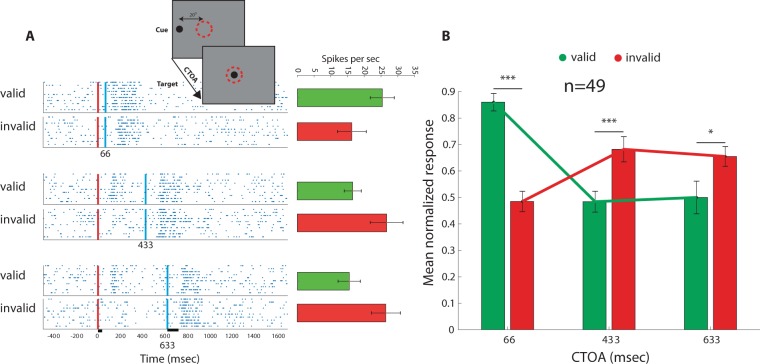
Tectal responses to the cueing paradigm**. (A)** An example from a single recording site. The inset shows an illustration of the paradigm in an invalid trial. The red dashed circle designates the position of the RF on the screen. A cue first apeears 20° away from the RF followed by a target in the RF. The rasters show the responses in 10 trials. The red vertical lines show the time of cue onset. The blue vertical lines show the times of target onset. The upper half of each raster shows the trials in which the cue was in the RF and the lower half, the trials in which the cue was outside the RF. Average responses and SEMs are shown in the column plot on the right. (**B)** The average normalized responses are shown for all three CTOAs. Mean responses to valid trials (green columns) are compared with mean responses to invalid trials (red columns). Error bars designate SEMs. One asterix indicates a p-value smaller than 0.05. Three asterixs indicate a p-value smaller than 0.001.

Multiunit responses were collected from 124 recording sites in the intermediate/deep layers of the right OT of four barn owls. We first replicated the time course of the cueing paradigm used in the behavioral experiments; a brief 33 ms cue was flashed on the screen. Later, with varying CTOA, a circular target appeared in the RF for 100 ms. The raster plots in Fig. 4A compare the responses of a single recording site to when the cue was inside the RF (valid) with the responses when the cue was outside the RF (invalid). In the short 66 ms CTOA, the average response in the invalid trials was lower compared to the valid trials, while it was enhanced in the longer CTOAs of 433 ms and 633 ms. These effects were significant at the group level (Fig. 4B). The strength of the response to the target in the RF in the valid trials switched from stronger in the short CTOAs to weaker in the long CTOAs, and vice versa in the invalid trials.

**Figure 5:**
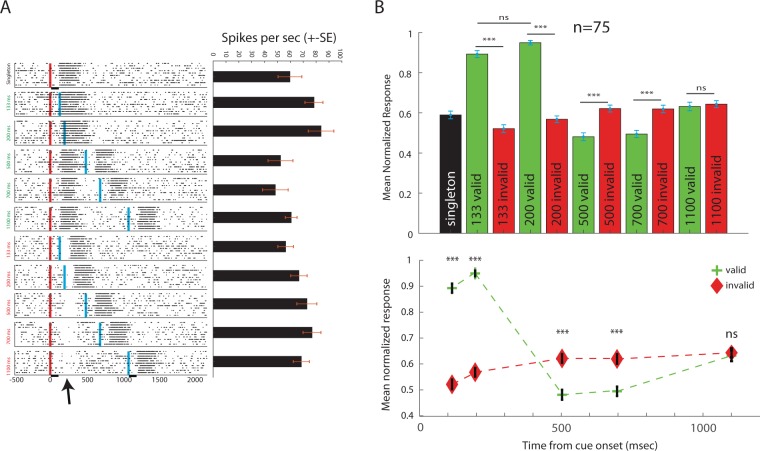
Tectal respopnses to non-overlapping stimuli. (**A**) An example of the responses of a single recording site. The upper raster plot shows responses of 10 trials to a single stimulus in the RF (singleton). The remaining 10 raster plots show responses to two non-overlapping stimuli. The red vertical line indicates the onset time of the first stimulus and the blue vertical line the onset time of the second stimulus. The time between the two stimuli (CTOA) varied from 133 ms to 1100 ms. The arrow points to a decrease relative to spontaneous firing observed in invalid trials. The vertical bars on the lower x-axis show the durations of the stimuli (100 ms). The bar plot on the right shows the average response in each of the corresponding conditions. (**B)** The population average of the normalized responses of 75 recording sites, tested with the paradigm shown in (**A**). Green columns show results from trials in which the first stimulus was in the RF (valid) and red columns from trials in which the first stimulus was outside the RF (invalid). The black column shows the average response to the singleton stimulus in the RF. Error bars designate SEMs. Vertical lines show results of the post-hoc Tukey test. Three asterixs designate a p-value smaller than 0.001. (**C)** The average response to the second stimulus in the RF as a function of the CTOA is compared between valid trials (green dashed line) and invalid trials (red dashed line).

The findings above demonstrate that the response properties of neurons in the OT agree with an IOR effect, i.e., suppressed responses to targets in a previously cued location. To explore this phenomenon further, we next used two identical stimuli of 100 ms duration. The stimuli were presented sequentially with varying CTOAs, either both inside the RF (valid), or the first outside the RF and the second inside the RF (invalid). The raster plots in Fig. 5A show an example of such an experiment. In the lower raster plots where the first stimulus was outside the RF, a drop in spontaneous activity can be seen following the first stimulus (arrow in Fig. 5A). The average of the means of 75 recording sites is shown in Fig. 5B,C. The pattern of response again shows a switch from facilitation to inhibition in the valid trials, and vice versa in the invalid trials (Fig. 5C, ANOVA, F (10,782) = 75.7, p < 0.001, post-hoc Tuckey tests p values shown in Fig. 5B). The average response to the invalid trials switched to stronger responses between a CTOA of 200 ms to 500 ms, and the difference between the invalid and valid trials disappeared at a CTOA of 1100 ms. Note, however, that in this experiment, the cue was 100 ms long. Therefore, to avoid overlap between the two stimuli, the CTOAs here were 67 ms longer than in the previous experiment, however, the interval between the two stimuli was kept the same in both experiments.

**Figure 6:**
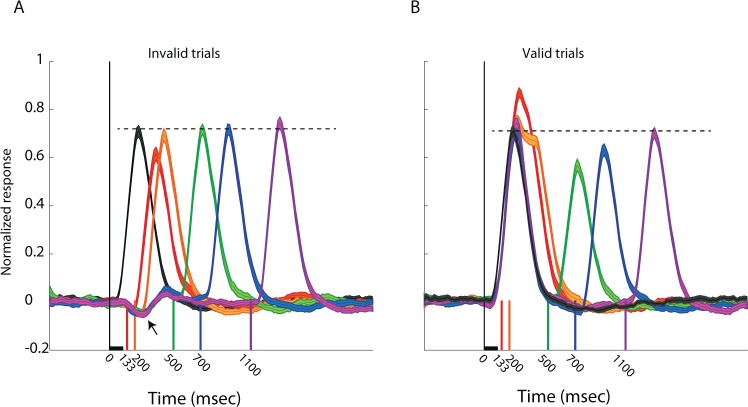
Population PSTHs showing the time course of the responses. (**A)** The population PSTH of the singleton response (black curve) is compared with the population PSTHs of the responses to the invalid trials, in the five CTOAs. The vertical lines indicate the onset of the stimulus in the RF relative to the presentation of the first stimulus outside the RF (CTOA). The colors of the vertical lines indicate the colors of the corresponding PSTH curves. The dashed line marks the level of the singleton response. The horizontal bar above the x-axis depicts the time and duration of the first stimulus. (**B)** Same as in A but showing the population PSTHs in the valid trials.

The smoothed population PSTHs are shown in Fig. 6 separately for the invalid trials (Fig. 6A) and the valid trials (Fig. 6B). A lateral inhibition dip can be seen in the average curves of the invalid trials (arrow in Fig. 6A). This dip corresponds with the time of the peak of the response to the singleton stimulus in the RF (black PSTH in Fig. 6A) and both are substantially longer than the duration of the stimulus (vertical bar in Fig. 6A,B). The prolonged lateral inhibition interacts with the response to the next coming stimulus at the shorter CTOA (133 ms) and to a lesser extent with the response to the stimulus arriving at a CTOA of 200 ms. Thus, the responses to the stimulus at the short CTOAs are reduced in the invalid trials. The opposite occurs in the valid trials (both stimuli in the RF, Fig. 6B): the elongation of response beyond the duration of the stimulus implies an overlap between the responses to the two (non-overlapping) stimuli at the short CTOA, giving rise to an enhanced response in the RF. This enhanced response is then switched to a suppression of the responses at CTOAs larger than 200 ms up to an interval of 1 s (Fig. 6B).

## Discussion

In humans and non-human primates, IOR has been observed in a variety of conditions and has been employed as a basic effect governing the allocation of attention^5^. The spot of attention is first attracted to a cued location (attentional capture^39,40^) followed by removal from that cued location (IOR^41^). The neural mechanisms guiding this shift of attention are unknown. Studying IOR effects in a variety of animal species can contribute to unravelling these attentional mechanisms. However, thus far studies of IOR in non-mammalian species have been scarce. A notable exception are two recent studies in archer fish that demonstrated behavioral effects consistent with IOR^20,21^.

The owls in this study were rewarded for a gaze shift to the target and not to the cue. However, this did not prevent them in some of the trials to shift their gaze away from the fixation point before the onset of the target. In primate studies of IOR, in most cases subjects are trained or instructed (in humans) to maintain fixation at the center until a target appears^37,38^. In our study, we therefore removed the trials from the analysis in which the owls’ gaze was not maintained near the fixation center (Fig. 2D,E). In the archer fish study^20^, the fish were also not trained to maintain fixation throughout the CTOA period. However, due to the nature of the motor response used to probe the effect in the fish (squirting water jets on a computer screen above the water), the responses were slow relative to the CTOAs, preventing them from happening before the target onset. In our case, where the behavioral report is a change of gaze to the target, abrupt changes of gaze before the target’s appearance were a source of bias. Indeed, when including all trials in the analysis, the average response times to the invalid cued targets tended to increase at the longer CTOAs (Fig. 2B,C).

In a previous study addressing IOR in pigeons^42^, it was shown that pigeons respond to previously pecked locations faster compared to novel locations. This is the opposite from what is expected in IOR, suggesting that pigeons lack such an effect. While it is possible that barn owls differ from pigeons in their basic visual search mechanisms, the experimental paradigm used by Gibson et al. (2005) differed in an important manner from ours, making it difficult to conclude species differences. In the pigeon study, the birds were trained to peck on the cue location before the appearance of the target. Pecking on the cue before the target may have an ethological logic but is different from the classical Posner’s paradigm used in primates, archer fish and now barn owls, where the animals are not expected to respond behaviorally to the cue^43^. It should be noted, however, that in some experimental conditions in humans, responding to cues did not preclude the generation of the IOR effect^44^. Moreover, the IOR effect was observed even when no cues were presented, and subjects completed free visual search tasks^45^. In a more recent behavioral study of visual search in pigeons, the birds were trained to respond to one target while ignoring a temporally asynchronous second target. The results of this study showed effects that were suggested to be explained by attentional capture and IOR^46^.

In the attentional literature, a cue can be endogenous or exogenous^47,48^. An exogenous cue reflexively captures the attention, whereas, an endogenous cue directs the attention to a certain location based on internal priors. Examples of endogenous cues are a centrally presented arrow or a cue that reliably predicts the rewarded location^49^. In humans and other primates, both endogenous and exogenous cues control attention allocation^47–51^, however, IOR effects are not triggered by purely endogenous cues^52,53^.

In a previous study it has been shown that birds can be primed to shift attention endogenously^54^. In this study, we used an exogenous cue, therefore, it is not known whether in owls IOR operates in endogenous attention shifts. Interestingly, IOR in an endogenous cuing task was shown in archer fish^21^, suggesting that both types of attentional shifts are fundamental in evolution.

The neural mechanisms underlying IOR are unknown. Several studies link IOR with the oculomotor system and suggest that the superior colliculus (SC) plays a major role in IOR generation^15,16,52–55^. The SC/OT is an evolutionary conserved structure^26,51,56,57^. Thus, we expect the barn owl’s OT to be involved as well. Here, we showed that average neural activity in the deep/intermediate layers of the OT is consistent with an IOR effect. A possible caveat in our study is that we recorded from head-fixed sedated animals. However, in a previous study, we showed that barn owls in the same partly anesthetized condition responded with pupil dilation to a novel auditory stimulus and lost the pupil response to the same stimulus when it was not novel^35^. Thus, the experimental conditions used here should be adequate to study basic attentional mechanisms.

Neurons in the intermediate/deep layers of the OT are organized to form a retinotopic map where the level of firing rate is assumed to represent the perceived saliency of the location in the RF^58,59^. This activity guides gaze shift (overt attention)^60,61^ and/or covert attention^35^ to the location of the most salient event. The sudden appearance of a short visual cue in the RF elicited a strong response that tended to continue for about 250 ms following termination of the stimulus (Fig. 6). A second visual stimulus appearing during this interval induced a response that interacted with the response to the previous stimulus, giving rise to an enhanced firing rate in the valid case (Fig. 6B). Such an enhanced firing rate is expected to attract gaze faster^34,62,63^. Following the initial phase of facilitation, a second phase of forward suppression occurred that lasted for about one second after the stimulus (Fig. 6B). Forward suppression or adaptation to excitation^64,65^ lasting for about one second has been reported in the owl’s OT^33^. Its origin is likely to involve synaptic suppression in upstream pathways^33^. Interestingly, forward suppression of the response in the monkey’s SC with the same time course as here has been associated with the IOR and was shown to be mediated by mechanisms upstream to the SC^28^. A subset of neurons in the monkey’s FEF was shown to respond preferentially to stimuli that had been fixated earlier in the trial^66^. Such neurons could provide top-down feedback to the OT to produce the IOR effect^67^.

A pattern of IOR can occur by forward facilitation of the neural response to the second stimulus in the RF followed by forward suppression at later CTOAs^68^. However, it can also occur by lateral suppression (caused by an invalid cue, i.e., a stimulus outside the RF) at short CTOAs, which dissipates at longer CTOAs. Here, we show that both occur in the responses of the OT. When a sudden cue appeared at a position outside the RF, lateral inhibition dominated the responses inside the RF. Lateral inhibition in the intermediate/deep layers of the OT is mostly carried by the isthmo-tectal loop^57,69,70^. This unique form of lateral inhibition is powerful and long-ranging^71^, and has been proposed as an efficient mechanism to select the strongest stimulus in the scene^59,72^. The circuitry has been studied thoroughly but within the context of competition between simultaneously appearing stimuli^73,74^. Here, we expose modulation by lateral inhibition of responses to asynchronous stimulation. We show that the lateral inhibition exceeded the termination of the stimulus by about 250 ms. Thus, our results show that the temporal pattern of lateral inhibition in the OT is suitable not only for stimulus competition but also for supporting attentional capture.

Following the time course of the cueing effect on the average neural responses in the OT (Fig. 4B), we expect to find a facilitation effect of RTs at the behavioral level at a CTOA of 66 ms and an inhibition effect at CTOAs of 433 and 633 ms. Both owls showed a tendency for facilitation of responses at a CTOA of 66 ms. However, the inhibitory effect was observed at a CTOA of 433 ms in one of the owls and at 633 ms in the other owl (Fig. 2D,E). This discrepancy between the patterns of average neural responses and behavioral responses may suggest that other brain areas are involved in the IOR. However, it could also reflect complications at the behavioral level that do not exist at the neuronal level, adding “noise” to the behavioral results. These may include additional, hard to control, cognitive processes apart from the IOR that affect behavioral responses, such as stress level, motivation and arousal. Indeed, variability between individuals is a common observation in the IOR effect in humans and animals^20,75^ and has also been manifested in the group of human subjects tested here. For example, some individuals in our experiment responded slower to the valid cued targets independent of the CTOA (Fig. 3B).

Previous studies have demonstrated that under certain conditions, inhibitory tagging of previously attended locations is attached to environmental coordinates rather than retinal coordinates^76,77^. The tectal map is retinotopic, thus, for IOR to occur in environmental coordinates, remapping of responses is required with every movement of the animal^78^. We do not know if IOR in barn owls is remapped to maintain location stability in the face of movements. However, neural responses in the OT are modulated by top-down projections^79,80^, moreover, in monkeys, remapping in the SC after saccades has been shown^81,82^. Thus, remapping of tectal responses according to head movements is a possibility. Another possibility is that the basic computation of IOR occurs in the OT but it is then relayed to higher brain areas for further processing. In primates, the prime source of remapping has been suggested to be outside the SC (in the parietal lobe)^83–85^.

In this study, we present results supporting an IOR effect in the barn owl. Together with previous studies on IOR in archer fish^20,21^, the results strengthen the hypothesis that the psychological effects of IOR, as well as attentional capture, are a manifestation of basic mechanisms expressed in the OT that evolved early in evolution to guide target selection and orientation^86^. The study stresses the importance of further comparative studies in a variety of animal species on the role of OT in guiding attention.

## Supplementary information

Supplementary video.

Supplementary spreadsheet.

## Data Availability

The data supporting the findings of this study are available from the corresponding author on reasonable request. The trial by trial data of all experiments in this study are available in the Supplementary spreadsheet.
